# Disentangling and quantifying the relative cognitive impact of concurrent mixed neurodegenerative pathologies

**DOI:** 10.1007/s00401-024-02716-y

**Published:** 2024-03-23

**Authors:** Carolina Maldonado-Díaz, Satomi Hiya, Raquel T. Yokoda, Kurt Farrell, Gabriel A. Marx, Justin Kauffman, Elena V. Daoud, Mitzi M. Gonzales, Alicia S. Parker, Leyla Canbeldek, Lakshmi Shree Kulumani Mahadevan, John F. Crary, Charles L. White, Jamie M. Walker, Timothy E. Richardson

**Affiliations:** 1 Department of Pathology, Molecular and Cell-Based Medicine, Icahn School of Medicine at Mount Sinai, Annenberg Building, 15.238, 1468 Madison Avenue, New York, NY 10029 USA; 2 Nash Family Department of Neuroscience, Icahn School of Medicine at Mount Sinai, New York, NY 10029 USA; 3 Neuropathology Brain Bank and Research CoRE, Icahn School of Medicine at Mount Sinai, New York, NY 10029 USA; 4 Department of Artificial Intelligence and Human Health, Icahn School of Medicine at Mount Sinai, New York, NY 10029 USA; 5 Ronal M. Loeb Center for Alzheimer’s Disease, Icahn School of Medicine at Mount Sinai, New York, NY 10029 USA; 6 Friedman Brain Institute, Icahn School of Medicine at Mount Sinai, New York, NY 10029 USA; 7 Department of Neurology, Icahn School of Medicine at Mount Sinai, New York, NY 10029 USA; 8 Department of Pathology, University of Texas Southwestern Medical Center, Dallas, TX 75390 USA; 9 Department of Neurology, Cedars Sinai Medical Center, Los Angeles, CA 90048 USA; 10 Department of Neurology, University of Texas Health Science Center at San Antonio, San Antonio, TX 78229 USA; 11 Glenn Biggs Institute for Alzheimer’s and Neurodegenerative Diseases, University of Texas Health Science Center at San Antonio, San Antonio, TX 78229 USA

**Keywords:** Alzheimer disease neuropathologic change, Limbic-predominant age-related TDP-43 encephalopathy, Lewy body dementia, Age-related tauopathy, Pick disease, Frontotemporal lobar dementia, Progressive supranuclear palsy, Corticobasal degeneration, Cerebrovascular disease, MMSE, CDR

## Abstract

Neurodegenerative pathologies such as Alzheimer disease neuropathologic change (ADNC), Lewy body disease (LBD), limbic-predominant age-related TDP-43 encephalopathy neuropathologic change (LATE-NC), and cerebrovascular disease (CVD) frequently coexist, but little is known about the exact contribution of each pathology to cognitive decline and dementia in subjects with mixed pathologies. We explored the relative cognitive impact of concurrent common and rare neurodegenerative pathologies employing multivariate logistic regression analysis adjusted for age, gender, and level of education. We analyzed a cohort of 6,262 subjects from the National Alzheimer’s Coordinating Center database, ranging from 0 to 6 comorbid neuropathologic findings per individual, where 95.7% of individuals had at least 1 neurodegenerative finding at autopsy and 75.5% had at least 2 neurodegenerative findings. We identified which neuropathologic entities correlate most frequently with one another and demonstrated that the total number of pathologies per individual was directly correlated with cognitive performance as assessed by Clinical Dementia Rating (CDR®) and Mini-Mental State Examination (MMSE). We show that ADNC, LBD, LATE-NC, CVD, hippocampal sclerosis, Pick disease, and FTLD-TDP significantly impact overall cognition as independent variables. More specifically, ADNC significantly affected all assessed cognitive domains, LBD affected attention, processing speed, and language, LATE-NC primarily affected tests related to logical memory and language, while CVD and other less common pathologies (including Pick disease, progressive supranuclear palsy, and corticobasal degeneration) had more variable neurocognitive effects. Additionally, ADNC, LBD, and higher numbers of comorbid neuropathologies were associated with the presence of at least one *APOE* ε4 allele, and ADNC and higher numbers of neuropathologies were inversely correlated with *APOE* ε2 alleles. Understanding the mechanisms by which individual and concomitant neuropathologies affect cognition and the degree to which each contributes is an imperative step in the development of biomarkers and disease-modifying therapeutics, particularly as these medical interventions become more targeted and personalized.

**Supplementary Information:**

The online version contains supplementary material available at 10.1007/s00401-024-02716-y.

## Introduction

Globally, the number of individuals living with dementia or some form of cognitive impairment is approximately 55–60 million individuals, but this is expected to increase approximately threefold by 2050 with an exponential rise in the yearly cost to patients, their families, and society at large [15, 31, 58, 68, 100]. Alzheimer disease (AD) neuropathologic change (ADNC) remains the most common underlying pathological finding in individuals with cognitive impairment, however it has become clear over the past decade that other neurodegenerative pathologies, including cerebrovascular disease (CVD), Lewy body disease (LBD), and limbic-predominant age-related TDP-43 encephalopathy neuropathologic change (LATE-NC), among others, are frequent comorbid findings [38, 56, 59, 63, 70, 75]. Recently, a number of studies have examined the cognitive effects of concomitant neuropathologies, and have suggested that a large percentage of cognitive impairment and dementia may be due to the additive or synergistic effects of comorbid disease states; however, it is unclear exactly how much each neurodegenerative disease contributes to overall cognitive decline and more specific cognitive and neuropsychological symptoms in individual patients or at the population level [3, 10, 11, 20, 22, 27, 29, 30, 34, 37, 38, 40, 45, 46, 53, 55, 56, 59–63, 70, 76, 84, 91, 95, 99]. There is evidence to suggest there are “normal levels” of common neurodegenerative pathologies at any given age, and the relatively recent concepts of “resistance” to developing neurodegenerative pathology with aging and cognitive “resilience” against the effects of pathology that is present have also been established [1, 51, 78, 93, 95]. There is also increasing evidence that “resilience” against a particular pathology may involve “resistance” to developing others; for example, a cognitively intact individual who is considered resilient against ADNC may have significantly less comorbid LATE-NC or CVD pathology compared to a cognitively impaired individual with similar levels of ADNC [1, 45, 50, 85].

A number of previous studies have focused on isolating individual disease processes in large cohorts to determine the specific cognitive contributions and other symptoms of a given (or combined) pathology [6–9, 13, 23, 25, 42, 91, 95]. This strategy is limited given the frequency with which many of these diseases co-occur and the relative scarcity of some isolated pathologies. This is particularly challenging in non-AD studies given the near ubiquity of some degree of ADNC findings in the aged population. Herein, we attempt to circumvent this issue using multivariate statistical models to disentangle and quantify the relative contributions of a number of common and rare neurodegenerative pathologies in 6,262 subjects with mixed pathologies from the National Alzheimer’s Coordinating Center (NACC) database. We evaluate the correlation between pathologies including ADNC, primary age-related tauopathy (PART), LBD, LATE-NC, hippocampal sclerosis, frontotemporal lobar degeneration with TDP-43 (FTLD-TDP), amyotrophic lateral sclerosis(ALS)/motor neuron disease (MND), Pick disease, progressive supranuclear palsy (PSP), corticobasal degeneration (CBD), and various forms of CVD (as well as additional covariates, including cerebral amyloid angiopathy (CAA), multiple system atrophy (MSA), chronic traumatic encephalopathy (CTE), and prion disease). In addition, we assess the cognitive impact of cumulative neurodegenerative pathologies, determine the amount of variation in cognition between subjects that can be directly attributed to each disease process, and determine the relative likelihood of impairment of global cognition and specific cognitive/neuropsychological domains (memory, attention, executive function, processing speed, and language) for each neurodegenerative pathology in an effort to determine the relative contribution of each individual pathology to cognitive impairment, irrespective of comorbid findings.

## Methods

### Case selection and exclusion criteria

Data for this study were downloaded with permission from the NACC (sourced from 37 ADRC collection centers located across the United States), which is a widely utilized cohort with available neuropathologic and neurocognitive data [7–9, 13, 23–28, 30, 39, 54, 67, 71, 72, 79–81, 91, 93, 95], established with funding from the National Institute on Aging (U01 AG016976) (https://naccdata.org/). We utilized standardized Uniform Data Set (UDS), version 3 variable definitions (https://naccdata.org/data-collection/forms-documentation/uds-3), Neuropathology (NP) Data Set, version 11 variable definitions (https://naccdata.org/data-collection/forms-documentation/np-11), and Genetic Data Set (Gen) variable definitions (https://files.alz.washington.edu/documentation/rdd-genetic-data.pdf) from NACC, as previously described [4, 5, 93]. A total of 6,262 unique NACC cases with global Clinical Dementia Rating (CDR®) Dementia Staging Instrument at the final clinical visit and recorded neuropathological autopsy data were identified for analysis.

### Neuropathologic, genetic, and demographic variables

Each neurodegenerative pathology was assessed from NACC variables. Where available, ADNC level was determined from the NACC NP dataset variable NPADNC. In instances where NPADNC was not available, ADNC levels were derived from a combination of Braak stage (NACCBRAA), Thal phase (NPTHAL), and CERAD neuritic plaque (NP) score (NACCNEUR) [12, 32, 52, 83]. A total of 4,137 cases had discernable ADNC levels (66.1% of all cases). Definite PART was assessed from a combination of NACCBRAA, NPTHAL, and NACCNEUR, and was defined here as Braak stage III-IV in the absence of diffuse or neuritic plaques in the neocortex (Thal phase 0 and CERAD NP score “none”) [17, 91, 96]. A total of 245 cases met these criteria for definite PART. LBD stage [47] was assessed using the NACC NP dataset variable NACCLEWY, which was available for 5,980 cases (95.5%).

FTLD-TDP, ALS/MND, and LATE-NC were assessed using NACC NP dataset variables NPFTDTDP, NPALSMND, NPTDPA (TDP-43 immunoreactive inclusions in the spinal cord), NPTDPB (TDP-43 immunoreactive inclusions in amygdala), NPTDPC (TDP-43 immunoreactive inclusions in the hippocampus), and NPTDPE (TDP-43 immunoreactive inclusions in neocortex). Cases with a neuropathologic diagnosis of FTLD-TDP and TDP-43 immunoreactive inclusions in the neocortex were included as FTLD-TDP. Cases were assigned LATE-NC stage 0 in the absence of TDP-43 immunoreactivity in any region, LATE-NC stage 1 with TDP-43 immunoreactive inclusions in the amygdala only, LATE-NC stage 2 with TDP-43 immunoreactive inclusions in the amygdala and hippocampus but not neocortex, and LATE-NC stage 3 with TDP-43 inclusions in the amygdala, hippocampus, and neocortex and an absence of a diagnosis of FTLD-TDP or ALS/MND [18, 30, 39, 43, 53, 55]. A total of 2,483 cases had sufficient data to determine FTLD-TDP status (39.7%), 2,960 cases had sufficient data to determine ALS/MND status (47.3%), and 1,916 cases had sufficient data to determine LATE-NC status (30.6%).

Hippocampal sclerosis was determined with the NACC NP dataset variable NPHIPSCL (n = 3,011; 48.1%). Pick disease was determined with the NACC NP dataset variable NACCPICK (n = 6,182; 98.7%). PSP was determined with the NACC NP dataset variable NACCPROG (n = 6,141; 98.1%). CBD was determined with the NACC NP dataset variable NACCCBD (n = 6,141; 98.1%). MSA was determined with the NACC NP dataset variable NPPDXB (n = 3,098; 49.5%). CTE was determined with the NACC NP dataset variable NPFTDT7 (n = 3,054; 48.8%). Prion disease was determined with the NACC NP dataset variable NACCPRIO (n = 6,067; 96.9%). CVD was determined using a combination of infarcts/lacunes (NACCINF; n = 6,217; 99.3%), hemorrhages/microbleeds (NACCHEM; n = 6,110; 97.6%), arteriolosclerosis (NACCARTE; n = 5,608; 89.6%), and white matter rarefaction (NPWMR; n = 2,757; 44.0%). CAA was determined with the NACC NP dataset variable NACCAMY (n = 6,116; 97.7%). Of note, CTE, MSA, and prion disease were used as covariates for multivariate logistic regression analysis, but are not displayed in figures.

Patient age at death was derived from the UDS variable NACCDAGE, patient sex was assessed with the UDS variable SEX, race was determined from the UDS variable RACE, and education was assessed with the UDS variable EDUC. Clinical assessment of normal cognition, mild cognitive impairment (MCI), or dementia was assessed with the UDS variable NACCUDSD. *APOE* genotype (ε2/2, ε2/3, ε2/4, ε3/3, ε3/4, ε4/4) were assessed with the variable NACCAPOE. Demographic, genetic, and pathologic data on all individuals included in this study can be found in Table 1.

**Table 1:** Demographic, genetic, and pathologic features

	All CDR	CDR = 0	CDR = 0.5	CDR = 1	CDR = 2	CDR = 3	p-value
n	6262	668	961	1068	1376	2189	−
Age (years)	80.0 ± 0.1	85.8 ± 0.4	84.3 ± 0.3	79.5 ± 0.4	79.1 ± 0.3	77.3 ± 0.3	** < 0.0001**
Gender (M:F)	3385:2877	271:397	537:424	643:425	791:585	1143:1046	** < 0.0001**
Education (years)	15.3 ± 0.1	15.7 ± 0.1	15.6 ± 0.1	15.1 ± 0.1	15.1 ± 0.1	15.2 ± 0.1	**0.0046**
*APOE* Status							
≥ 1 *APOE* ε2 allele	11.1% (607)	17.4% (110)	14.6% (127)	12.9% (119)	9.5% (116)	7.3% (135)	** < 0.0001**
≥ 1 *APOE* ε4 allele	44.0% (2416)	19.7% (124)	31.7% (276)	44.2% (408)	49.4% (605)	54.6% (1003)	** < 0.0001**
Total number of pathologies	1.93 ± 0.01	0.87 ± 0.03	1.43 ± 0.03	1.86 ± 0.03	2.31 ± 0.03	2.19 ± 0.02	** < 0.0001**
ADNC							
Not	9.9% (408)	23.3% (79)	15.8% (84)	10.5% (70)	5.1% (50)	7.7% (125)	** < 0.0001**
Low	13.1% (544)	40.7% (138)	22.2% (118)	12.0% (80)	10.1% (99)	6.7% (109)	
Intermediate	14.1% (582)	29.8% (101)	25.4% (135)	14.9% (99)	10.3% (10)1	9.0% (146)	
High	62.9% (2603)	6.2% (21)	36.5% (194)	62.6% (416)	74.4% (726)	76.6% (1246)	
Braak stage							
0	6.9% (426)	5.4% (36)	5.5% (52)	8.7% (91)	8.1% (74)	8.1% (173)	** < 0.0001**
I	8.2% (505)	19.9% (132)	10.9% (103)	7.0% (73)	7.2% (82)	6.2% (115)	
II	10.5% (646)	27.0% (179)	16.4% (155)	10.3% (108)	6.7% (87)	5.5% (117)	
III	10.1% (620)	24.1% (160)	15.8% (149)	10.6% (111)	7.9% (98)	4.8% (102)	
IV	12.9% (795)	17.1% (113)	22.4% (212)	15.9% (166)	11.5% (155)	6.9% (149)	
V	17.8% (1091)	5.1% (34)	15.3% (145)	19.6% (205)	23.0% (310)	18.5% (397)	
VI	33.5% (2061)	1.2% (8)	13.6% (129)	27.8% (291)	40.1% (540)	50.9% (1093)	
Thal phase							
0	13.7% (418)	23.7% (79)	18.9% (89)	13.8% (71)	8.1% (53)	11.7% (126)	** < 0.0001**
1	8.8% (268)	20.7% (69)	11.1% (52)	6.6% (34)	7.2% (47)	6.2% (66)	
2	5.8% (178)	10.5% (35)	8.9% (42)	5.% (29)	6.7% (44)	2.6% (28)	
3	11.6% (353)	20.1% (67)	14.5% (68)	13.0% (67)	7.9% (52)	9.2% (99)	
4	18.4% (559)	18.0% (60)	20.0% (94)	20.6% (106)	19.2% (126)	16.1% (173)	
5	41.7% (1268)	6.9% (23)	26.6% (125)	43.2% (207)	50.8% (333)	54.1% (580)	
CERAD NP score							
None	23.5% (1462)	47.4% (316)	31.7% (303)	25.7% (274)	17.1% (234)	15.4% (335)	** < 0.0001**
Sparse	23.7% (791)	23.4% (156)	19.6% (188)	10.9% (116)	10.2% (139)	8.8% (192)	
Moderate	18.6% (1161)	18.3% (122)	22.9% (219)	20.2% (215)	17.1% (234)	17.1% (371)	
Frequent	45.2% (2818)	10.8% (72)	25.8% (247)	43.2% (460)	55.7% (762)	58.7% (1277)	
Definite PART (Braak III-IV)	4.0% (245)	10.4% (69)	6.3% (60)	4.0% (42)	2.0% (27)	2.2% (47)	** < 0.0001**
Lewy body stage							
None	68.8% (4114)	83.9% (547)	74.4% (694)	67.4% (700)	64.4% (841)	64.8% (1332)	** < 0.0001**
Brainstem	3.9% (231)	5.4% (35)	5.9% (55)	3.9% (41)	3.1% (40)	2.9% (60)	
Limbic	14.3% (857)	7.7% (50)	10.4% (97)	14.8% (154)	16.3% (213)	16.7% (343)	
Neocortical	13.0% (779)	3.1% (20)	9.3% (87)	13.8% (143)	16.1% (210)	15.5% (319)	
LATE-NC stage							
0	70.3% (1347)	88.6% (156)	77.4% (229)	72.4% (249)	61.0% (280)	67.6% (433)	** < 0.0001**
1	7.2% (137)	2.8% (5)	6.1% (18)	8.1% (28)	9.2% (42)	6.9% (44)	
2	17.8% (341)	6.8% (12)	13.9% (41)	14.8% (51)	23.5% (108)	20.1% (129)	
3	4.7% (91)	1.7% (3)	2.7% (8)	4.7% (16)	6.3% (29)	5.5% (35)	
Hippocampal sclerosis	13.9% (420)	2.7% (9)	8.2% (37)	15.0% (76)	17.8% (114)	17.1% (184)	** < 0.0001**
FTLD-TDP	6.4% (159)	1.5% (4)	2.5% (10)	6.9% (31)	5.5% (32)	10.5% (82)	** < 0.0001**
ALS/MND	1.7% (49)	3.1% (10)	1.7% (8)	2.2% (11)	2.4% (15)	0.5% (5)	**0.0036**
Pick’s disease	1.6% (101)	0.3% (2)	0.4% (4)	1.4% (15)	1.5% (21)	2.8% (59)	** < 0.0001**
PSP	4.1% (221)	1.1% (7)	5.8% (55)	4.7% (50)	2.7% (37)	3.4% (72)	** < 0.0001**
CBD	1.9% (121)	0.5% (3)	2.0% (19)	1.8% (19)	2.1% (28)	2.4% (52)	**0.0343**
CTE	0.6% (17)	0.3% (1)	0.4% (2)	0.8% (4)	1.0% (7)	0.3% (3)	0.2617
Prion disease	1.5% (90)	0.2% (1)	0.8% (7)	2.7% (28)	1.2% (16)	1.8% (38)	**0.0001**
Gross infarcts	18.6% (158)	18.1% (121)	24.3% (231)	20.7% (218)	16.4% (223)	16.8% (365)	** < 0.0001**
Gross hemorrhage	6.6% (407)	6.1% (42)	8.3% (77)	6.8% (70)	6.4% (85)	6.2% (133)	0.2434
Arteriolosclerosis							
None	20.2% (1134)	22.0% (128)	19.8% (172)	20.5% (204)	19.8% (248)	19.9% (382)	**0.0002**
Mild	35.8% (2007)	43.2% (251)	37.1% (322)	36.5% (362)	34.1% (426)	33.7% (646)	
Moderate	30.8% (1730)	26.7% (155)	29.7% (258)	31.5% (313)	31.1% (389)	32.1% (615)	
Severe	13.1% (737)	8.1% (47)	13.4% (116)	11.5% (114)	15.0% (188)	14.2% (272)	
White matter rarefaction							
None	41.7% (1149)	45.3% (146)	45.6% (183)	48.5% (217)	40.2% (231)	36.7% (372)	** < 0.0001**
Mild	28.4% (784)	33.9% (109)	29.2% (117)	27.5% (123)	32.6% (187)	24.5% (248)	
Moderate	19.5% (537)	12.7% (41)	17.5% (70)	17.2% (77)	18.3% (105)	24.1% (244)	
Severe	10.4% (287)	8.1% (26)	7.7% (31)	6.7% (30)	8.9% (51)	14.7% (149)	
CAA							
None	40.9% (2499)	59.0% (386)	49.9% (469)	41.4% (432)	34.1% (463)	35.3% (749)	** < 0.0001**
Mild	28.1% (1717)	27.2% (178)	26.7% (251)	29.4% (307)	30.8% (417)	26.6% (564)	
Moderate	19.9% (1219)	10.2% (67)	14.1% (132)	19.6% (205)	23.2% (315)	23.5% (500)	
Severe	11.1% (682)	3.5% (23)	9.3% (87)	9.6% (100)	11.9% (161)	14.6% (311)	

### Cognitive and neuropsychological variables

Representative cognitive and neuropsychological variables encompassing overall cognition and specific neuropsychological domains were assessed using the UDS variables. These included global CDR (CDRGLOB; n = 6,262; 100%), CDR Sum of Boxes (CDRSUM; n = 6,262; 100%), Mini-Mental State Examination (MMSE; NACCMMSE; n = 3,548; 56.7%), logical memory immediate recall (LMI) (LOGIMEM; n = 2,778; 44.4%), logical memory delayed recall (LMD) (MEMUNITS; n = 2,735; 43.7%), digit span forward (DSF) (DIGIF; n = 2,867; 45.8%), digit span backward (DSB) (DIGIB; n = 2,828; 45.2%), Trail Making Test Part A (TMT-A) (TRAILA; n = 2,604; 41.6%), Trail Making Test Part B (TMT-B) (TRAILB; n = 1,870; 29.9%), Wechsler Adult Intelligence Scale Digit Symbol Substitution Test (WAIS DS) (WAIS; n = 1,970; 31.5%), animal fluency (ANIMALS; n = 3,310; 52.9%), vegetable fluency (VEG; n = 3,190; 50.9%), and Boston Naming Test, 30 odd items (BNT) (BOSTON; n = 2,725; 43.5%), as previously described [7, 27, 28, 30, 36, 80, 91]. The total number of subjects with available data for each neuropathologic feature and cognitive test combination is available in Supplemental Table 1.

Each cognitive/neuropsychological test was adjusted for age, sex, and education level as previously described [73, 91, 98]. The neurocognitive tests (LMI, LMD, DSF, DSB, TMT-A, TMT-B, WAIS DS, animals, vegetables, and BNT) were converted into z-scores and the percentile was determined for each with corrections for age, sex, and education, where “mild impairment” was defined as the 2–8.99 percentile, “moderate impairment” was defined as the 1–1.99 percentile, and severe impairment was defined as < 1 percentile [73]. We defined impairment for the purposes of multivariate logistic regression analysis as < 9th percentile, including mild-severe impairment. Global CDR was defined as impaired using both 0.5 and 1 as thresholds. CDR sum of boxes (SOB) was defined as impaired using both 3 and 4.5 as thresholds. MMSE was defined as impaired using scores of 21 and 24 as thresholds. All figures and tables presented here display data using global CDR ≥ 1, CDR SOB ≥ 4.5, and MMSE ≤ 24 [2, 21, 57, 64, 88].

### Data analysis

Multivariate logistic regression analysis was performed with MedCalc (MedCalc Software Ltd, Ostend, Belgium). All other statistical analyses were performed with GraphPad Prism version 9.5.1 (GraphPad Software, Inc., La Jolla, CA, USA). All graphs were created using GraphPad Prism; graphs of linear regression analysis between cognitive status (global CDR, CDR SOB, and MMSE) and the total number of neurodegenerative pathologies were created as composites, combining multiple variable bubble plots and linear regression analysis, where the size of each data point represents the number of subjects. Differences in the proportion of gender, *APOE* status, and neuropathologic variables among cohorts were calculated using Fisher’s exact test. Differences between age, education, and total number of pathologies between groups were evaluated using multiple t-tests. Correlations with total number of pathologies were modeled using linear regression and Pearson correlation coefficient. Percent contributions of each pathology to cognitive impairment was determined using multiple regression analysis where the calculated β coefficient for each pathology was divided by the sum of all β values, as previously described in detail [10, 66]. Statistical significance was set at α = 0.05.

## Results

### Demographic features of the cohort as a whole

There were a total of 6,262 individuals with available CDR, with score groups ranging from 0 to 3 (Table 1). CDR score of 0 represents an individual with no cognitive impairment. CDR scores of 0.5, 1, 2, and 3 represent individuals with questionable, mild, moderate, and severe cognitive impairment, respectively. Clinically, 13.7% of the total cohort were not impaired at the last clinical visit, while 10.4% had MCI, 74.1% had dementia, and 1.8% were impaired but did not meet criteria for either MCI or dementia. The average age for the general cohort was 80 years old (80.0 ± 0.1 for all CDR). There was a statistically significant difference in age among CDR score groups that highlights the relationship between cognitive impairment severity and mortality, where age was inversely correlated to overall CDR, suggesting that subjects with the most severe disease may die earlier, or perhaps there is a degree of selective attrition. The average age for individuals with no cognitive impairment (CDR = 0) was 85.8 ± 0.4 years old, while the average age for individuals with severe cognitive impairment (CDR = 3) was 77.3 ± 0.3 years old (p < 0.0001). Gender was predominantly male in groups with questionable to severe cognitive impairment (55.7% male in CDR 0.5–3), but cognitively intact individuals were significantly more likely to be female (59.4% female in CDR = 0 group) (p < 0.0001). The average level of education was 15.3 ± 0.1 years, and individuals with no cognitive impairment display a modest but statistically significant higher level of education when compared to cognitively impaired individuals (15.7 ± 0.1 years for CDR = 0 compared to 15.2 ± 0.1 years for CDR = 3; p = 0.0046). For the entire cohort (all CDR), 607 individuals (11.1%) have ≥ 1 *APOE* ε2 allele and 2,416 individuals (44.0%) have ≥ 1 *APOE* ε4 allele. The frequency of *APOE* ε4 alleles was positively correlated with CDR; 19.7% of subjects with CDR = 0 had at least one *APOE* ε4 allele, while 54.6% of subjects with CDR = 3 had at least one *APOE* ε4 allele (p < 0.0001). Conversely, the frequency of *APOE* ε2 alleles was inversely correlated with CDR; 17.4% of subjects with CDR = 0 had at least one *APOE* ε2 allele, while only 7.3% of subjects with CDR = 3 had at least one *APOE* ε2 allele (p < 0.0001) (Table 1).

### Pathologic features of the cohort as a whole

In subjects with at least ADNC, LATE-NC, LBD, and cerebrovascular pathology data available (n = 1,847), the total number of neuropathologic findings was close to 2 per individual (1.93 ± 0.01 for all CDR). 95.7% of individuals had at least one identified neuropathologic finding at autopsy, and 75.5% had at least two neuropathologic findings. The number of pathologies per individual was directly correlated with global CDR; there were 0.87 ± 0.03 neuropathologic diagnoses in cognitively intact subjects (CDR = 0) compared to 2.19 ± 0.02 neuropathologic diagnoses in subjects with severe cognitive impairment (CDR = 3; p < 0.0001) (Table 1).

**Fig. 1:**
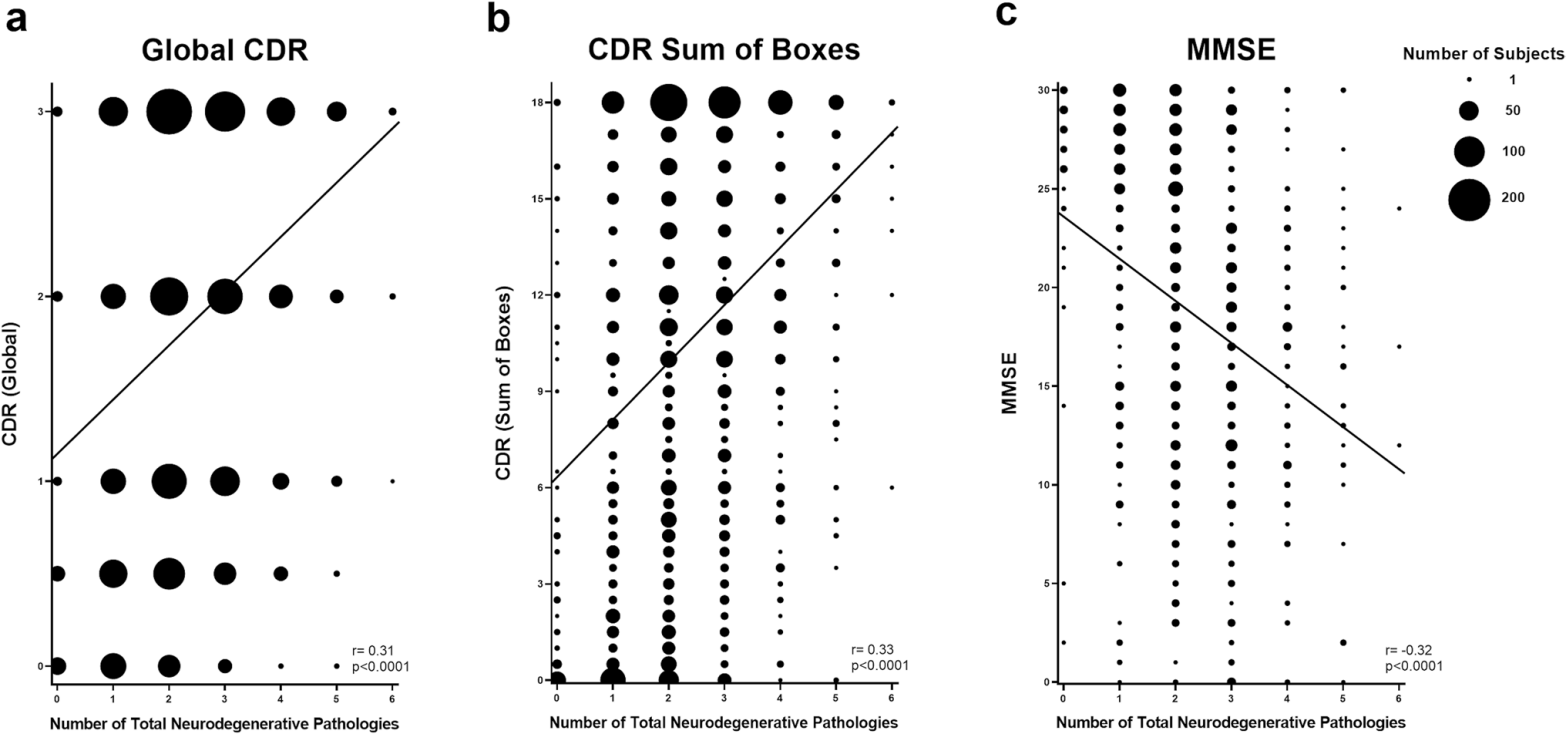
Linear regression analysis demonstrating strong correlation between the total number of neuropathologic variables identified at autopsy (including ADNC, PART, LBD, LATE-NC, hippocampal sclerosis, FTLD-TDP, ALS/MND, Pick disease, PSP, CBD, CVD, CTE, prion disease, AGD, and MSA) and (**a**) global CDR, (**b**) CDR sum of boxes, and (**c**) MMSE (size of each data point corresponds to the number of subjects)

The number of neurodegenerative pathologies identified at autopsy was proportional to measures of global cognition by linear regression analysis. There was a positive correlation between the number of total neurodegenerative pathologies and the global CDR (r = 0.31, p < 0.0001) (Fig. 1a), which assesses memory, orientation, judgement, and problem solving, as well as functioning across community affairs, home and hobbies, and personal care domains [57, 64]. Similarly, there was a linear relationship between the number of total neurodegenerative pathologies and CDR sum of boxes (r = 0.33, p < 0.0001) (Fig. 1b). MMSE includes tests assessing orientation, memory, attention/concentration, naming, verbal repetition and comprehension, reading and writing, and visuospatial abilities. A perfect score is 30 points, while a score of less than 25 is consistent with cognitive impairment [2, 21]. There was an inverse relationship between number of total neurodegenerative pathologies and MMSE (r = -0.32, p < 0.0001) (Fig. 1c). These same trends were present in patients with intermediate or high level ADNC; there was a direct correlation between the number of *additional* pathologies co-occurring in patients with intermediate or high level ADNC and global CDR (r = 0.20, p < 0.0001) and CDR sum of boxes (r = 0.21, p < 0.0001), and an indirect correlation with MMSE (r = −0.15, p = 0.0012). Taken together, these data demonstrate that individuals with higher numbers of concurrent pathologies generally have more severe levels of cognitive impairment as measured by CDR and MMSE.

Compared to cognitively intact subjects, individuals with moderate and severe cognitive impairment were found to have higher levels of certain pathologies like ADNC (74.4% of CDR 2 and 76.6% of CDR 3 subjects had high level ADNC compared to only 6.2% of CDR 0 subjects; p < 0.0001), limbic and neocortical stage LBD (p < 0.0001), hippocampal and neocortical stage LATE-NC (p < 0.0001), hippocampal sclerosis (p < 0.0001), FTLD-TDP (p < 0.0001), infarcts (p < 0.0001), arteriolosclerosis (p = 0.0002), white matter rarefaction (p < 0.0001), and CAA (p < 0.0001), while the frequency of definite PART decreased (p < 0.0001) (Table 1). The frequency of ALS/MND was also inversely proportional to cognitive decline (p = 0.0036), which may be due to these patients succumbing to the non-cognitive components of their illness before developing TDP-43-associated cognitive impairment.

### Features of cognitively intact individuals

Interestingly, many cognitively normal individuals (CDR scores of 0) displayed some degree of pathology, and in a minority of cases very severe levels of individual pathologies and co-morbid neuropathologies. Individuals with no cognitive impairment averaged less than one neurodegenerative finding at autopsy (Table 1); however, a small percentage of individuals exhibited significant neuropathologic changes. Among those with no cognitive impairment (n = 668), 101 and 21 individuals had intermediate and high level ADNC respectively, 50 and 20 individuals had limbic and neocortical stage LBD respectively, 12 and 3 had LATE-NC stage 2 and 3 respectively, 9 had hippocampal sclerosis, 4 had FTLD-TDP, 2 had Pick disease, 10 had ALS/MND, 121 had gross infarcts, 42 had gross hemorrhage, 47 had severe arteriolosclerosis, 26 had severe white matter rarefaction, and 23 had severe CAA [82, 87, 97]. Only 17.3% of subjects with a CDR of 0 had no identifiable pathology, while 82.7% had at least one significant neuropathologic finding and 42.8% had at least 2 concurrent neurodegenerative pathologies, with up to 5 of the assessed neurodegenerative findings identified in one individual with a CDR of 0 and MMSE of 30, suggesting a significant level of resilience in the face of neurodegenerative pathology in a minority of subjects.

### Correlation between neurodegenerative pathologies

**Fig. 2:**
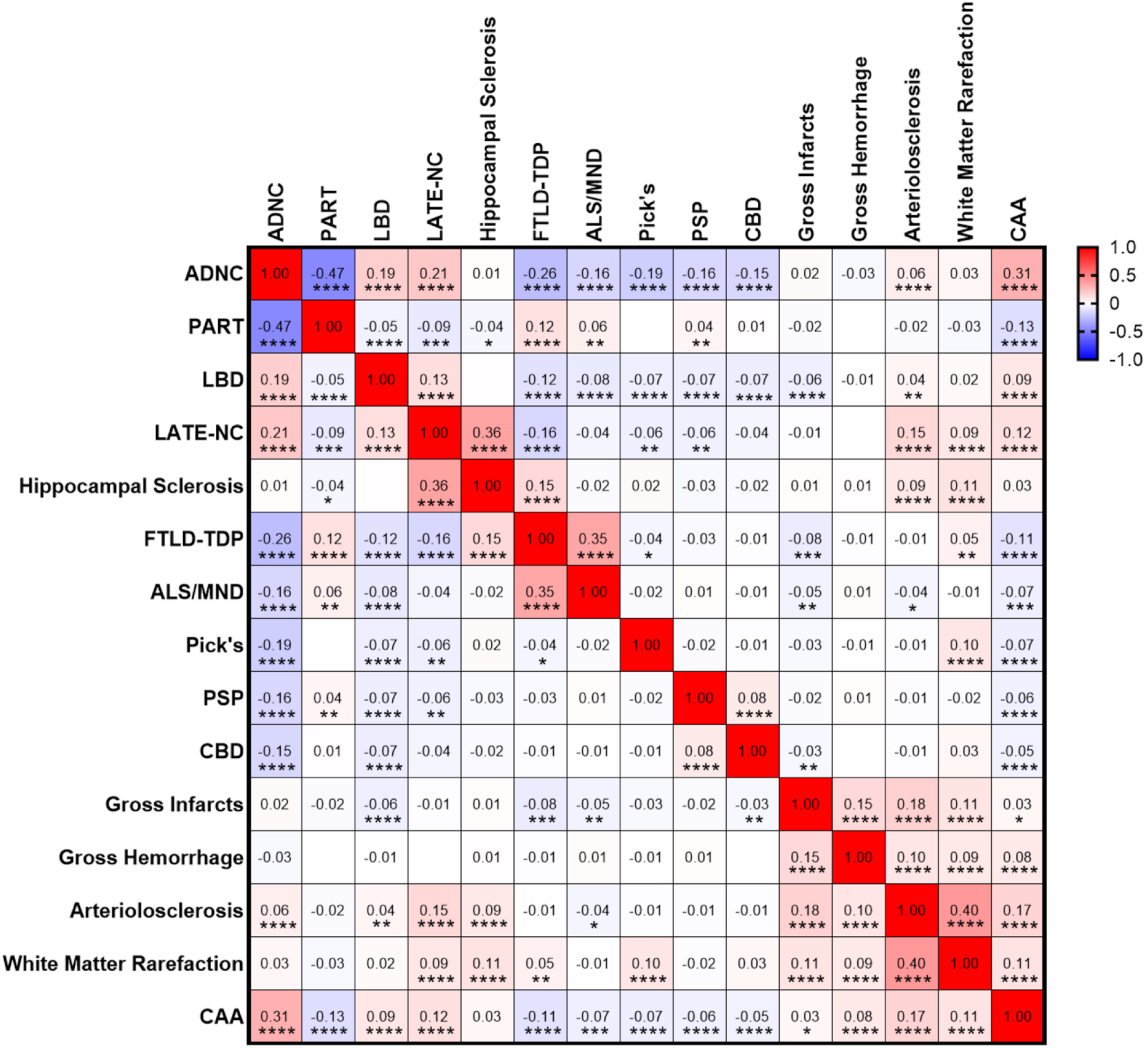
Correlation matrix assessing different autopsy-proven neuropathologic findings across 6,262 subjects in the NACC dataset. * p < 0.05; ** p < 0.01; *** p < 0.001; **** p < 0.0001

In this cohort, some neurodegenerative pathologies correlated more frequently with others. For example, ADNC showed a high correlation with CAA (Pearson r = 0.31; p < 0.0001), LATE-NC (r = 0.21; p < 0.0001), LBD (r = 0.19; p < 0.0001), and arteriolosclerosis (r = 0.06; p < 0.0001). In contrast, ADNC was negatively correlated with FTLD-TDP (r = −0.26; p < 0.0001), Pick disease (r = -0.19; p < 0.0001), ALS/MND (r = −0.16; p < 0.0001), PSP (r = -0.16; p < 0.0001), and CBD (r = -0.15; p < 0.0001). By definition, ADNC was inversely correlated with definite PART (r = −0.47; p < 0.0001) since the diagnosis of definite PART requires the absence of β-amyloid [17, 52, 96]. Hippocampal sclerosis was significantly correlated with TDP-43 pathologies (both LATE-NC and FTLD-TDP), as well as arteriolosclerosis and white matter rarefaction, but not other neurodegenerative pathologies, including ADNC [19, 26, 30, 39]. Individual cerebrovascular pathologies tended to correlate with one another, and in particular arteriolosclerosis was highly correlated with white matter rarefaction (r = 0.40; p < 0.0001) (Fig. 2). Braak stage, Thal phase, CERAD neuritic plaque density, diffuse plaque density, CAA, and arteriolosclerosis were all significantly correlated, while hippocampal sclerosis was again most correlated to TDP-43 and, to a lesser extent, vascular variables (Supplemental Fig. 1).

### Univariate and multivariate analysis of cognitive impact of comorbid neuropathologies

Due to the frequency with which subjects (particularly those with greater levels of cognitive impairment) had more than one neurodegenerative pathology (Table 1 and Fig. 1), and the relative frequency with which some neuropathologic findings (ADNC, LATE-NC, LBD, CVD) tended to co-occur (Fig. 2), we explored which neuropathologies contributed to global and specific aspects of cognitive impairment [61]. To unravel which of these pathologies were associated with varying severities of cognitive impairment, we examined all variables by performing multivariate logistic regression analysis after adjusting each individual test for age, gender, and years of education [63, 73].

**Fig. 3:**
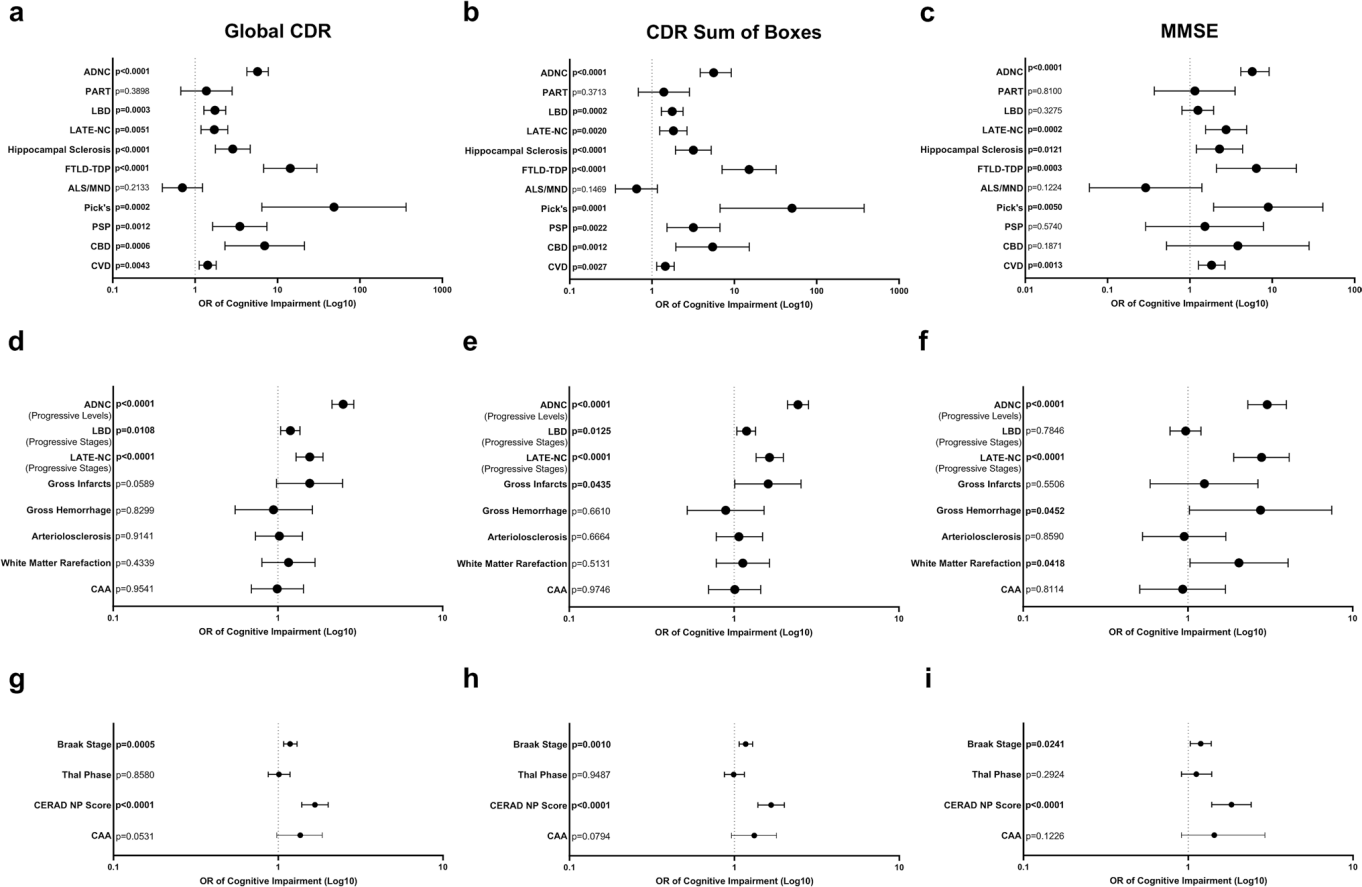
Multivariate logistic regression analysis demonstrating the odds ratios (OR) and 95% confidence intervals of cognitive impairment in the presence of ADNC (level 2–3), definite PART (Braak III-IV), LBD (stage 2–3), LATE-NC (stage 2–3), hippocampal sclerosis, FTLD-TDP, ALS/MND, Pick disease, PSP, CBD, and CVD in terms of (**a**) global CDR, (**b**) CDR sum of boxes, and (**c**) MMSE. CTE, MSA, and prion disease were included as covariates in multivariate analysis model but are not shown here for simplicity. Multivariate logistic regression analysis performed on progressive levels/stages of ADNC, LBD, LATE-NC, infarcts, hemorrhage, arteriolosclerosis, white matter rarefaction, and cerebral amyloid angiopathy in terms of (**d)** global CDR, (**e**) CDR sum of boxes, and (**f**) MMSE. Multivariate logistic regression analysis was also performed on progressive Braak stage, Thal phase, CERAD NP score, and CAA (with LBD, LATE-NC, and CVD used as covariates) in terms of (**g**) global CDR, (**h**) CDR sum of boxes, and (**i**) MMSE

Neuropathologic, cognitive, and neuropsychological variables were studied to analyze the contribution of each pathology to impairment of overall cognition, as well as impairment of specific neuropsychological domains. In terms of global CDR (using a threshold of CDR = 1), the presence of ADNC (combined intermediate and high level), LBD (combined stage 2–3), LATE-NC (combined stage 2–3), hippocampal sclerosis, FTLD-TDP, Pick disease, PSP, CBD, and CVD all demonstrated a significant and independent risk of cognitive impairment (Fig. 3a). The multivariate odds ratio for cognitive impairment of individuals with level 2 or 3 ADNC was 5.72 (4.25–7.72 95% CI; p < 0.0001) (Supplemental Table 2), indicating that individuals with level 2 or 3 ADNC were 5.72 times more likely to experience cognitive impairment when compared to individuals without this pathology, due to the contribution of ADNC alone (i.e., regardless of comorbid pathologies). The multivariate odds ratio for cognitive impairment for individuals with Pick disease was 48.45 (6.47–362.62 95% CI; p = 0.0002), with FTLD-TDP was 14.30 (6.79–30.12 95% CI; p < 0.0001), with CBD was 6.98 (2.30–21.21 95% CI; p = 0.0006), with PSP was 3.49 (1.63–7.44 95% CI; p = 0.0012), with hippocampal sclerosis was 2.86 (1.76–4.65 95% CI; p < 0.0001), with stage 2 or 3 LBD was 1.74 (1.28–2.35 95% CI; p = 0.0003), with stage 2 or 3 LATE-NC was 1.71 (1.18–2.49 95% CI; p = 0.0051), and with CVD was 1.42 (1.12–1.80 95% CI; p = 0.0043). Similar results were seen for CDR sum of boxes using 4.5 as a threshold for cognitive impairment (Fig. 3b). These same interactions were also seen using global CDR = 0.5 and CDR sum of boxes = 3.0 as thresholds.

For MMSE, the multivariate odds ratio for cognitive impairment was significant for ADNC, LATE-NC, hippocampal sclerosis, FTLD-TDP, Pick disease, and CVD (Fig. 3c). The multivariate odds ratio for cognitive impairment for individuals with intermediate or high level ADNC was 5.72 (3.58–9.15 95% CI; p < 0.0001), for individuals with stage 2 or 3 LATE-NC was 2.75 (1.55–4.88 95% CI; p = 0.0002), with hippocampal sclerosis was 2.29 (1.20–4.36 95% CI; p = 0.0121), with FTLD-TDP was 6.41 (2.10–19.64 95% CI; p = 0.0011), with Pick disease was 8.93 (1.94–41.23 95% CI; p = 0.0050), and with CVD was 1.83 (1.27–2.66 95% CI; p = 0.0013). Taken together, these results demonstrate that ADNC, LATE-NC, CVD, hippocampal sclerosis, Pick disease, and FTLD-TDP significantly impact overall cognition as independent variables, as evidenced by a poor cognitive performance in terms of global CDR, CDR sum of boxes, and MMSE (Fig. 3a-c). LBD, PSP, and CBD were found to independently impact cognitive performance as measured by global CDR and CDR sum of boxes but this association was not found with MMSE. These findings highlight the relative contribution of each neuropathology to overall cognition and specific neuropsychological domains (Supplemental Table 2).

Multivariate logistic regression analysis was also performed to evaluate the effects of progressive levels of the most commonly encountered neurodegenerative findings, including ADNC, LATE-NC, LBD, and cerebrovascular disease to increase the granularity of these findings. Progressive levels of ADNC had an odds ratio of cognitive impairment of 2.49 (2.13–2.89 95% CI; p < 0.0001) for global CDR (Fig. 3d and Supplemental Table 3), each progressive stage of LBD had an odds ratio of 1.19 (1.04–1.36 95% CI; p = 0.0108, and each progressive stage of LATE-NC had an odds ratio of 1.56 (1.29–1.88 95% CI; p < 0.0001). In contrast, specific cerebrovascular pathologies (infarcts, hemorrhage, arteriolosclerosis, white matter rarefaction) and CAA did not have a significant contribution to cognitive impairment as examined by global CDR. In general, similar results are seen for CDR sum of boxes (Fig. 3e), apart from the fact that gross infarcts displayed a significant contribution to cognitive impairment with an odds ratio of 1.61 (1.01–2.55 95% CI; p = 0.0435). For MMSE, the multivariate odds ratio for cognitive impairment was significant for progressive levels of ADNC, progressive stages of LATE-NC, the presence of hemorrhage and white matter rarefaction (Fig. 3f). The multivariate odds ratio for cognitive impairment for progressive levels of ADNC was 3.03 (2.31–3.96 95% CI; p < 0.0001), for progressive stages of LATE-NC was 2.80 (1.90–4.12 95% CI; p < 0.0001), for gross hemorrhage was 2.76 (1.02–7.47 95% CI; p = 0.0452), and for white matter rarefaction was 2.04 (1.03–4.06 95% CI; p = 0.0418). We also performed an analysis of the impact of individual components of ADNC [52]. Using multivariate logistic regression analysis (with CVD, LATE-NC, and LBD as covariates) we identified progressive Braak stage and CERAD NP score as significantly affecting the global CDR, CDR sum of boxes, and MMSE, while progressive Thal phase and presence of moderate-severe CAA were not significantly associated with cognitive impairment (Fig. 3g–i).

**Fig. 4:**
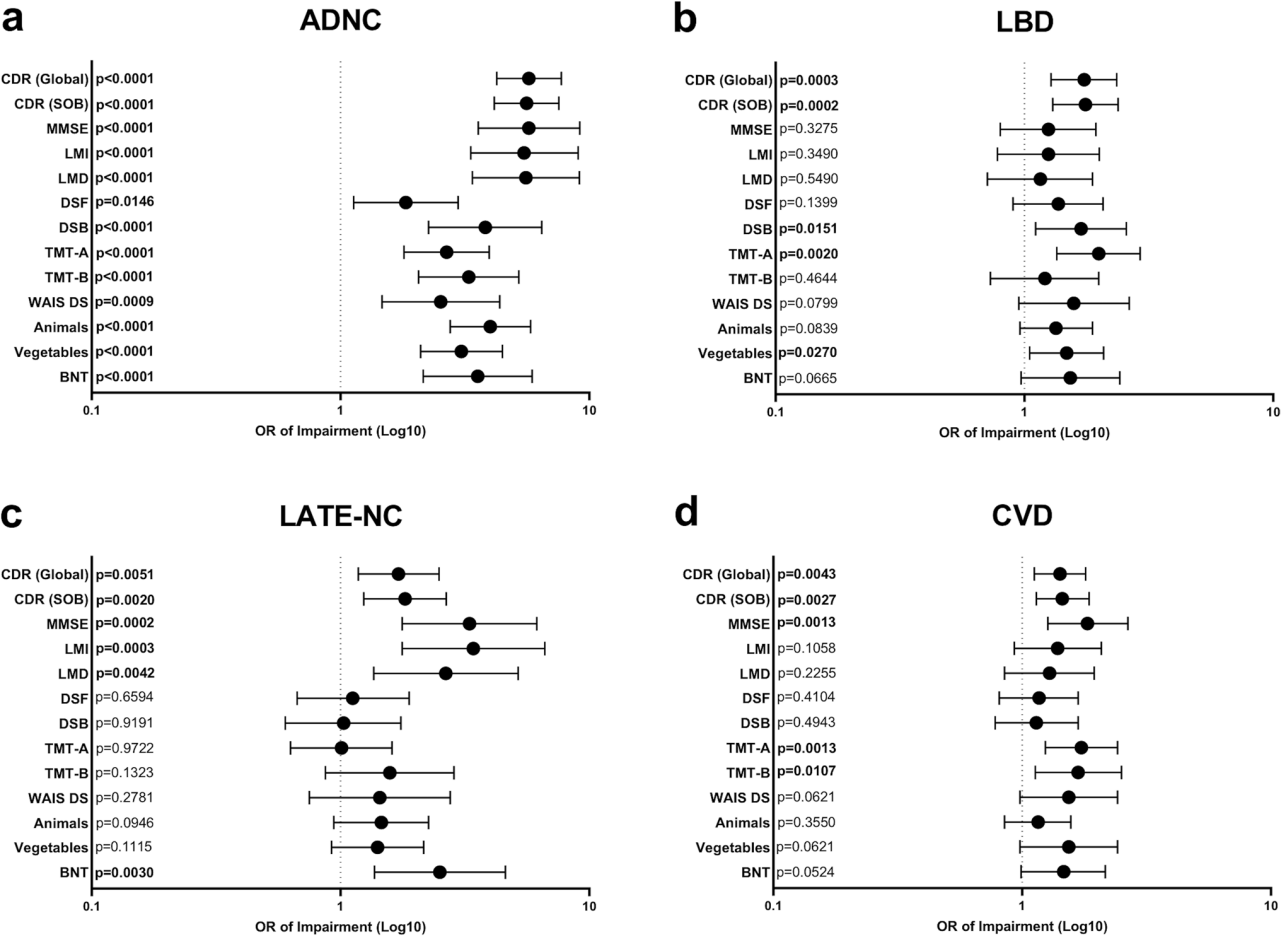
Multivariate logistic regression analysis demonstrating the odds ratios (OR) and 95% confidence intervals of impairment for global CDR, CDR sum of boxes, MMSE, logical memory immediate recall (LMI), logical memory delayed memory (LMD), digit span forward (DSF), digit span backward (DSB), trail making test A (TMT-A), trail making test B (TMT-B), Wechsler adult intelligence scale digit symbol substitution (WAIS DS), animal fluency (Animals), vegetable fluency (Vegetables), and Boston naming test (BNT) in the four most commonly identified neuropathologies, (**a**) ADNC, (**b**) LBD, (**c**) LATE-NC, and (**d**) CVD (including infarcts/lacunes, hemorrhages/microhemorrhages, moderate-severe arteriolosclerosis, and moderate-severe white matter rarefaction). Significance and odds ratios for each cognitive/neuropsychological test was determined in the context of ADNC, PART, LBD, LATE-NC, hippocampal sclerosis, FTLD-TDP, ALS/MND, Pick disease, PSP, CBD, CVD, CTE, prion disease, AGD, and MSA

Using multiple regression analysis, we determined that the four most common neuropathologic features (ADNC, LATE-NC, LBD, and CVD) explained 42.2–58.4% of the variation in global cognitive impairment between subjects as measured by CDR and MMSE, with ADNC explaining the majority of this variation (21.5–31.5%). LBD explained an additional 3.2–6.1% of variation in cognitive impairment, LATE-NC explained 7.1–14.5%, and CVD as a pooled group explained 6.9–10.2%; all other neuropathologic entities each explained ≤ 3% of variation in cognitive function when included in the model (Supplemental Fig. 2). Additionally, we performed multivariate logistic regression analysis on more specific cognitive/neuropsychological domains [30, 73, 98]. ADNC significantly affected all assessed cognitive domains (memory, attention, executive function, processing speed, and language), while LBD affected some domains related to attention, processing speed, and language (DSB, TMT-A, and vegetable naming), LATE-NC primarily affected tests related to logical memory and language domains (LMI, LMD, and BNT), while CVD only independently affected TMT-A and TMT-B tests (Fig. 4a-d and Supplemental Table 2). Furthermore, FTLD-TDP significantly impacted all tests related to logical memory, attention, and language domains (with additional effects in some measurements of processing speed), and Pick disease significantly affected a subset of tests associated with these same domains (Supplemental Table 2). Isolating cases with only the most common pathologies (ADNC, LBD, LATE-NC, and CVD) demonstrated significant effects across all domains for each progressive level of ADNC and impairment of logical memory and language domains for progressive stages of LATE-NC with less consistent associations with LBD and cerebrovascular disease (Supplemental Table 3).

### Impact of *APOE* status on each disease process

**Fig. 5:**
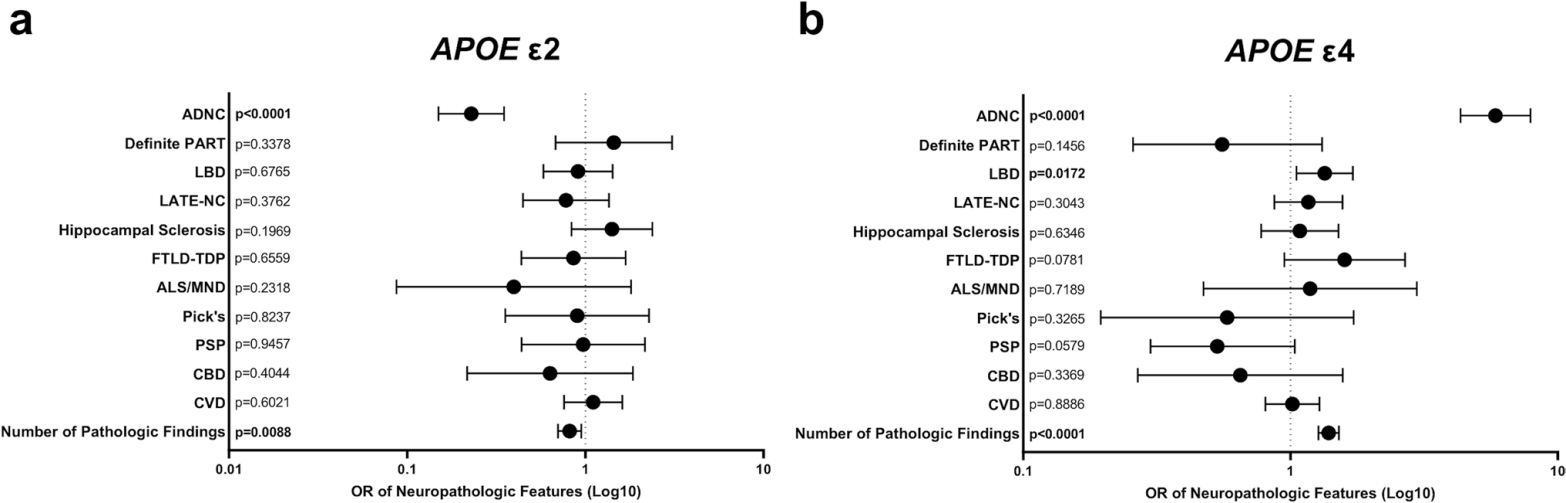
Multivariate logistic regression analysis demonstrating the odds ratios (OR) and 95% confidence intervals of an individual having (**a**) at least one *APOE* ε2 allele or (**b**) at least one *APOE* ε4 allele with the presence of each pathology: ADNC (level 2–3), definite PART (Braak III-IV), LBD (stage 2–3), LATE-NC (stage 2–3), hippocampal sclerosis, FTLD-TDP, ALS/MND, Pick disease, PSP, CBD, and CVD, as well as an increasing number of total pathologic findings

Using multivariate logistic regression analysis, we assessed the relationship between the presence of *APOE* ε2 and *APOE* ε4 alleles and each individual neurodegenerative process, as well as with the total number of neuropathologic features identified at autopsy. The presence of at least one *APOE* ε2 allele was inversely correlated with intermediate-high level ADNC (0.23 OR; 0.15–0.35 95% CI; p < 0.0001) and increasing numbers of total neuropathologies (0.82 OR; 0.70–0.95 95% CI; p = 0.0088) (Fig. 5a). The presence of at least one *APOE* ε4 allele was significantly associated with the presence of intermediate-high level ADNC (5.85 OR; 4.33–7.91 95% CI; p < 0.0001), limbic and neocortical stage LBD (1.34 OR; 1.05–1.71 95% CI; p = 0.0172), and increasing numbers of total neuropathologies (1.39 OR; 1.27–1.52 95% CI; p < 0.0001) (Fig. 5b). No significant interaction was noted between any other neurodegenerative disease process and the presence of either *APOE* allele.

## Discussion

Dementia is one of the leading causes of morbidity and mortality in the elderly population worldwide, with a significant anticipated rise in prevalence in coming decades [15, 58]. Alzheimer disease remains the most common underlying pathology associated with dementia, however it has recently become clear that many cases of dementia that are attributed to clinical Alzheimer disease have a variety of other comorbid neurodegenerative pathologies at the time of autopsy which may be responsible for some of the cognitive symptoms [3, 22, 25, 34, 37, 38, 40, 45, 56, 59–61, 63, 70, 76, 84, 85, 95, 99]. Given the degree of overlap among neurodegenerative diseases, particularly ADNC, LBD, LATE-NC, and various forms of CVD, it has been difficult to determine the exact contribution of each pathologic finding to an individual patient’s cognitive status, or particular cognitive/neuropsychological test scores. Moreover, established concepts such as resilience against Alzheimer disease pathology must be considered in the context of a wider array of neurodegenerative diseases, as this resilience may be related in part to a relative lack of comorbidities (i.e., *resilience* against Alzheimer disease pathology may be related to an individual’s *resistance* to developing comorbid TDP-43 or vascular pathology) [1, 45, 61, 63, 93, 95]. It is important to understand the biology underlying these individual and concomitant neuropathologies, as well as their additive and synergistic clinical effects, as the development of biomarkers, preventative measures, and both symptomatic and disease-modifying therapeutics will depend on an accurate and complete assessment of all factors contributing to cognitive impairment, particularly as these medical interventions become more personalized and targeted toward specific neuronal populations and protein accumulations [59, 85].

To this end, we analyzed a cohort of 6,262 subjects from the NACC database, ranging from 0–6 comorbid neuropathologic entities in individual patients, using multivariate logistic regression analysis to help unravel the relative contributions of ADNC, CAA, PART, LBD, LATE-NC, hippocampal sclerosis, FTLD-TDP, ALS, Pick disease, PSP, CBD, and CVD. As expected, the average number of neurodegenerative findings increases from less than one per cognitively intact subject to more than 2 per subject with moderate-severe cognitive impairment, and there is a direct correlation between cognitive impairment and the progressive level of common pathologies such as ADNC, LATE-NC, LBD, CVD, and CAA and frequency of rarer pathologies such as FTLD-TDP, Pick disease, PSP, CBD, while the frequency of definite PART decreases with increasing global CDR (Table 1). We found significant correlations between many of these pathologies (Fig. 2) and a direct correlation between the number of pathologies and cognitive impairment (Fig. 1). ADNC was the only underlying neurodegenerative pathology that significantly impaired all neuropsychological and cognitive domains as an independent variable (Fig. 4a), although notably many of the individual cognitive domains were more affected by other pathologies, in particular FTLD-TDP and Pick disease, which had greater effects than ADNC in many measures. A number of other neurodegenerative pathologies were significantly associated with more selective deficits (Supplemental Table 2). These results also demonstrate that Braak stage and CERAD NP score are the important determinants of cognitive impairment in ADNC, while Thal phase is not correlated with cognitive status (Fig. 3g-i) [72]. The presence of ADNC and increased numbers of neurodegenerative pathologies were inversely correlated with *APOE* ε2, while ADNC, LBD, and increased neurodegenerative pathologies were positively correlated with *APOE* ε4 (Fig. 5), suggesting that *APOE* status has minimal impact on non-ADNC neurodegenerative processes in isolation, but may play a role in the development of multiple concurrent proteinopathies [62, 95].

We did not find any significant cognitive impairment associated with definite PART (Supplemental Table 2) and definite PART was found more frequently in patients with lower global CDR scores (Table 1). This was similar to our previous findings in pure PART [91], although those demonstrated some isolated effects on processing speed, executive function, and visuospatial function, which were not found in the present study. This is consistent with previous observations that cognitive impairment in PART is correlated more with the overall hippocampal p-tau burden (as opposed to Braak stage), the presence of white matter pathology, and other comorbidities, including LATE-NC and CVD [7, 33, 44, 48, 74, 90, 91, 96]. This also supports the idea that definite PART is a separate process from ADNC, and may represent more of a normal aging pattern [16, 17, 35, 90, 94, 96]. The frequency of ALS/MND was also inversely correlated with global CDR (Table 1), which may be explained by subjects dying of ALS-related complications earlier than subjects without ALS, before more severe cognitive impairment from associated FTLD-TDP could develop. Another interesting finding is that hippocampal sclerosis is significantly associated with cognitive impairment, apparently independent of TDP-43 pathology (Fig. 3a-c). 81 cases of hippocampal sclerosis did not have a concurrent diagnosis of FTLD-TDP or LATE-NC (19.3% of total cases with hippocampal sclerosis) and in 9 cases (2.1%) hippocampal sclerosis was the only pathology identified, and these 9 cases had significant cognitive impairment (global CDR of 1.7 and MMSE of 19.6). This may be due to a wider range of underlying causes of CA1 neuron loss in the hippocampus, including epilepsy and severe global hypoxic-ischemic injury, two etiologies excluded from our earlier studies [30].

There are also a number of cases in which there was an apparent mismatch between the severity of pathology identified and the cognitive status (Fig. 1 and Table 1). 122 cognitively intact patients had intermediate or high level ADNC (36% of CDR = 0 patients with ADNC data available), 70 had limbic or neocortical LBD (10.8%), 15 had stage 2 or 3 LATE-NC (8.5%), 4 had FTLD-TDP (1.5%), and 2 had Pick disease (0.2%), among other pathologies. Perhaps most interestingly, only 17.3% had no significant neuropathologic findings, while 42.8% had 2 or more, and 1 subject had 5 pathologies (high level ADNC, LBD stage 2, LATE-NC stage 2, hippocampal sclerosis, and CVD). These data suggest that a subset of these cases are individuals who are resilient against one or more pathologies, a population which warrants additional study as there may be underlying biological differences that are unassessed with routine neuropathologic diagnosis [92]. There are also rare cases with a CDR score of 2–3 that lack any significant identified neuropathologic diagnoses. These cases may represent subjects with underlying pathologies that were unassessed due to previous versions of the NACC NP dataset, subjects with very low levels of multiple different pathologies adding up to produce a cognitive effect (i.e., low level ADNC in combination with LATE-NC stage 1, LBD stage 1, and/or relatively mild cerebrovascular changes), pathologic findings that do not fit into one or more of the designated NACC categories, or subjects with unspecified/undocumented genetic alterations [22]. Similar to previous studies [10, 66], between 42.2% and 58.4% of the variance in cognitive impairment was accounted for by the most common neuropathological findings (ADNC, LATE-NC, LBD, and CVD) (Supplemental Fig. 2). This suggests that the development of successful therapies with the capacity to remove or prevent any of these pathologies would remove a significant portion of the dementia burden from a given population. For example, successful treatment of CVD could eliminate up to 10% of cognitive impairment from the population as a whole, while a successful treatment of ADNC could potentially eliminate 30% of cognitive impairment [14].

There are a number of limitations associated with this study. While the study is based on a large patient population (total n = 6,262 subjects), all subjects are drawn from the NACC dataset, which is not necessarily representative of the population at large [69]. The NACC dataset is enriched for subjects with Caucasian ancestry, high levels of education, rare diseases/pathologies, more frequent *APOE* ε4 alleles, more severe dementia, and more severe neuropathologic findings, which may be related to population-specific selection and recruitment biases, including enrolling a higher number of patients with existing dementia compared to cognitively normal individuals [24, 86]. The variables included in both the clinical and neuropathological datasets have also undergone numerous revisions, and autopsy data on TDP-43 and Thal phase were not included until relatively recently with the NACC NP dataset version 10 [13]. The provided data for many variables include only the presence of regional pathology and in some cases the general distribution without severity/density/burden of pathology, making distinction between FTLD-TDP and LATE-NC difficult in some instances [55]. Most variables do not take into account bilateral pathologic features, which may be important as pathologic asymmetry may provide a source of cognitive reserve or resilience against certain pathologies, and may result in deficits to specific cognitive/neuropsychological domains [41, 49, 65, 77, 89]. While this study may not be fully representative of the relationship between mixed pathologies and cognition in the population at large as a result of these limitations, the methods employed here may serve as a framework which can be applied in additional clinic- and community-based cohorts to better elucidate the relative effects of each of these neurodegenerative processes individually and in combination.

In the context of the existing literature, our findings are consistent with the hypothesis that the additive effects of multiple pathologies may be responsible for a large portion of cognitive impairment experienced by elderly subjects. These results suggest that ADNC is the most common and most consistent factor affecting all cognitive domains, while others (including LATE-NC, LBD, and CVD) are more selective in their cognitive effects, and some frontotemporal dementias (FTLD-TDP, Pick disease, PSP, CBD) may have greater effects in some specific cognitive domains than ADNC. Given current trend toward developing personalized therapies and treatments designed to target specific protein aggregates and neuronal subtypes and populations, there is a critical need for the development of in vivo biomarkers that can accurately distinguish between neuropathologic processes (and progression/severity within processes), as well as distinguish which processes underlie specific cognitive symptoms, and which are modifiable [85]. The data presented in this report offer a step toward determining the relative effects of many of these disease processes and how they may interact, which is critical for accurate clinical diagnosis, as well as biomarker and drug development.

### Supplementary Information

Below is the link to the electronic supplementary material.

Supplementary file1 (TIF 1199 kb)

Supplementary file2 (TIF 3489 kb)

Supplementary file3 (XLSX 11 kb)

Supplementary file4 (XLSX 15 kb)

Supplementary file5 (XLSX 13 kb)

## Data Availability

The data presented in this manuscript are derived from the National Alzheimer’s Coordinating Center (NACC) dataset, and are available upon request from https://naccdata.org/.
