# Supplementary figures and images for: Disentangling and quantifying the relative cognitive impact of concurrent mixed neurodegenerative pathologies
Source: Acta Neuropathol. 2024 Mar 23;147(1):58. doi: 10.1007/s00401-024-02716-y (PMC10960766; doi:10.1007/s00401-024-02716-y)

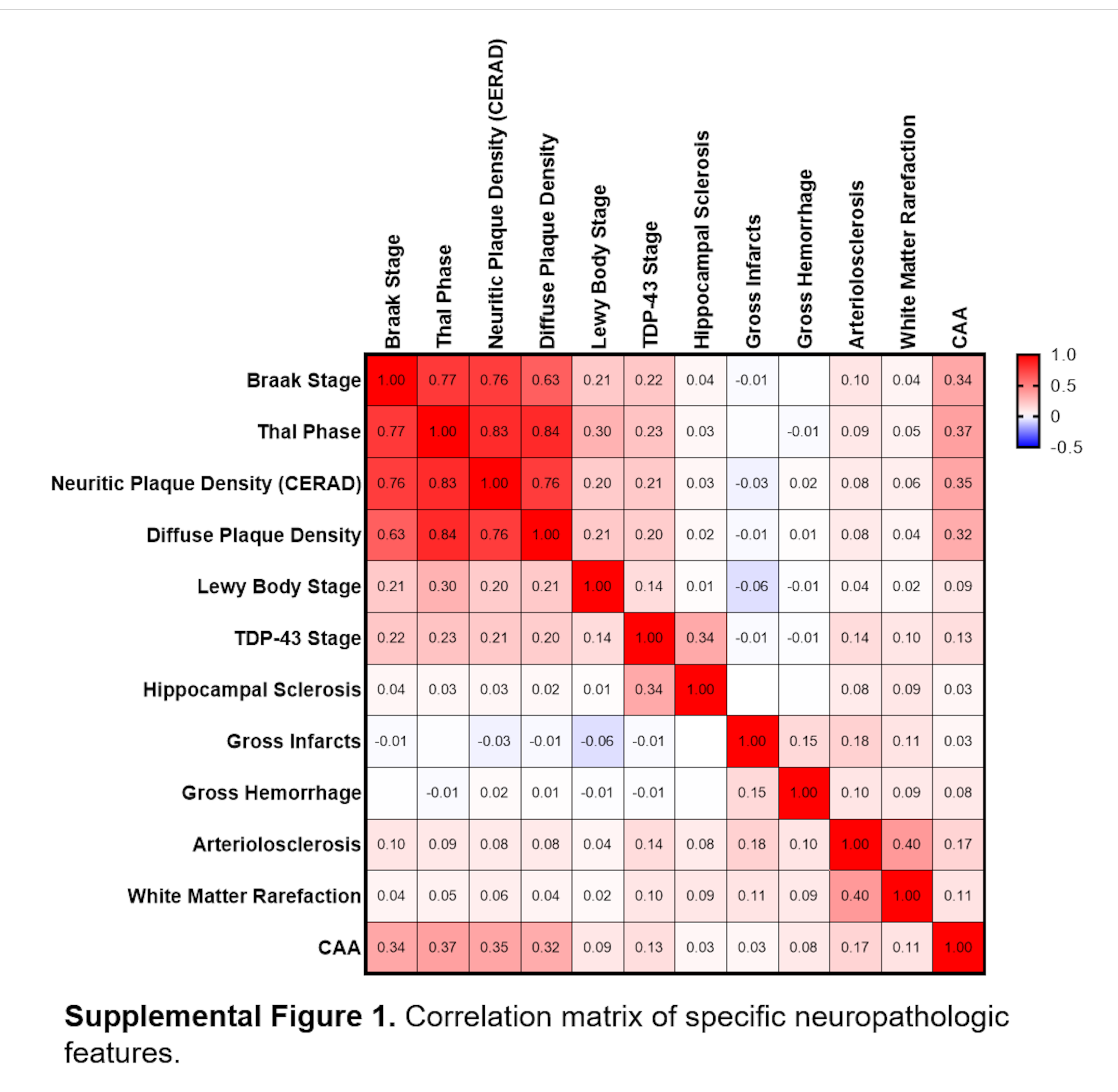

Supplement: Supplementary file 1 — Supplementary file1 (TIF 1199 kb) [file 401_2024_2716_MOESM1_ESM.tif]

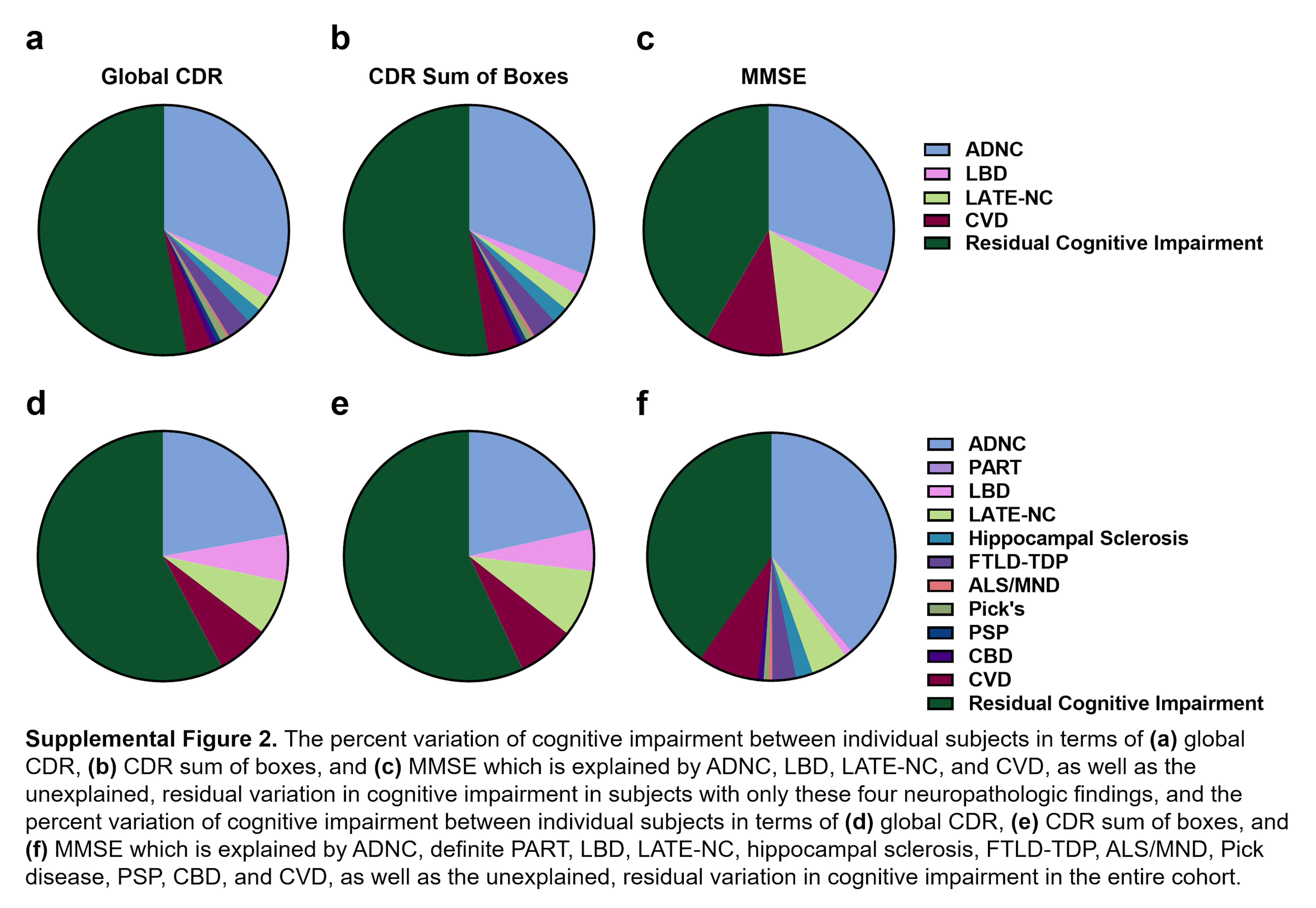

Supplement: Supplementary file 2 — Supplementary file2 (TIF 3489 kb) [file 401_2024_2716_MOESM2_ESM.tif]
